# Comparison of three sperm selection methods for ICSI-DGC, Cumulus column, and incubation with supernatant product of adipose tissue-derived adult stem cells: An experimental study

**DOI:** 10.18502/ijrm.v19i1.8184

**Published:** 2021-01-25

**Authors:** Zeynab Yazdanpanah, Mitra Heydari Nasrabadi, Zeynab Piravar

**Affiliations:** ^1^Department of Biology, Faculty of Science, Islamic Azad University Central Tehran branch, Tehran, Iran.; ^2^Department of Biology, Faculty of Biology Science, Islamic Azad University, Parand Branch, Tehran, Iran.

**Keywords:** Sperm, DNA fragmentation, Cumulus oophorus column, SPAS.

## Abstract

**Background:**

The examination of sperm parameters and sperm DNA integrity are necessary for male fertility expression. These parameters can be affected by method of sperm separation.

**Objective:**

To measure the damage of each sperm separation method on the sperm parameters and sperm DNA integrity.

**Materials and Methods:**

In this experimental study, semen samples of 20 infertile men with asthenoteratozoospermia (Infertility Research Center, Qom, Iran, 2017) were processed in three ways: density gradient centrifugation (DGC), cumulus column, and incubation with supernatant products of adipose tissue-derived adult stem cells (SPAS). The results of sperm parameters and DNA fragmentation before and after the process were statistically analyzed.

**Results:**

The number of separated sperms by normal morphologies during the SPAS and the cumulus column was significantly more than the corresponding population in the DGC group. In addition, although all three methods have the same ability to increase total sperm motility and the number of recovered sperms, in the field of forwarding movement and DNA fragmentation, the SPAS method performed more efficiently (p = 0.021).

**Conclusion:**

Sperm parameters and DNA fragmentation in the SPAS group were better than those in the DGC and cumulus column groups. Furthermore, it has been shown that the sperm capacity was increased with the SPAS method. However, the rearrangement of sperm chromatin by reducing the disulfide bridges and providing the possibility of re-histone over capacity causes a significant reduction in DNA fragmentation.

## 1. Introduction 

Infertility is clinically defined as the absence of pregnancy after by a couple after one year of regular unprotected sexual intercourse. Since sperm production disorders are the most common cause of male infertility, it is pertinent to recognize and correct these disorders to help infertile couples (1).

The effect of special preparation techniques for increasing the fertility rate of infertile couples under assisted reproductive technology (ART) is unclear. In the in vitro fertilization, normal spermatozoa should be isolated. Processes that are carried out should be safe, affordable, and cost-effective (2). The first preparation methods based on the simple separation of sperm from the seminal fluid were constructed through the washing technique (3). Subsequent methods were based on sperm density (mass/volume) to select motile and live sperm with normal morphology. These methods are mainly known as density gradient centrifugation (DGC) methods (4).

Moreover, semen analysis can only determine the sperm parameters and cannot reveal other aspects of sperm function such as DNA health and the sperm's ability to react with the oocyte. Therefore, an effective method for the sperm selection for ICSI is required, based on nuclei maturation, cytoplasm, and sperm membrane (5). DNA damage in mature spermatozoa can be due to defects in the chromatin compaction, resulting from either endogenous fracture in the DNA or during the process of apoptosis before the ejaculation. In addition, high levels of reactive oxygen species (ROS) production can also lead to DNA damage. In addition, environmental and hormonal factors and testis temperature increase are other potential causes of damage to sperm DNA. Considering the ill effects of DNA fragmentation, we determined the amount of damage to sperm DNA using three different methods of sampling of semen for the recruitment in a variety of ARTs, such as Intrauterine inseminatio) and Intracytoplasmic sperm injection (ICSI). Accordingly, we were able to detect the method that causes the least damage.

## 2. Materials and Methods 

### Sampling

This experimental study was performed on 20 infertile men with asthenoteratozoospermia referred to the unit of ACECR Infertility Research Center, Qom, Iran (Rooya) in 2017. Semen samples were taken from all participants after three-five days of intercourse. A part of the semen was used to measure the sperm parameters based on the 2010 WHO criteria (6). The percentage of DNA fracture was evaluated, and the remains were used to conduct the procedure preparations for supernatant products of adipose tissue-derived adult stem cells (SPAS), DGC, and cumulus pill.

The inclusion criteria were: sperm number > 20 million/ml, at least 3% normal morphology, and sperm motility rate between 40 and 65%. The exclusion criteria, on the other hand, were: high prevalence of agglutination in sample and varicoceles.

### Evaluation of sperm DNA damage

In this study, the sperm DNA fragmentation assay KIT (Pars iGene Co., Iran) was used to investigate the rate of DNA failure. This Kit is based on the Sperm Chromatin dispersion technique. Accordingly, washed and diluted semen samples were used for the Sperm Chromatin dispersion test, which was carried out according to a procedure by Fernandez (7). Slides were stained with Wright's stain (Merck 1.01383.0500). Sperm nuclei without fragmented DNA exhibited large- or medium-sized halos, whereas those with fragmented DNA appeared with small halos, no halos and solidly stained cores, or no halos and irregular or faintly stained cores (degraded).

### Sample preparation by DGC method 

Semen samples were subjected to the DGC. Briefly, sperm was prepared by standard DGC using 45% and 90% isolate (Irvine Scientific; Santa Ana, CA, USA). Only spermatozoa migrating in the lower plate were selected.

### Sample preparation by SPAS method 

The abdomen fat tissue samples were cut into small pieces and rinsed with PBS and transferred to a spa bath of 37°C for 1.5-3 hr. Next, the tubes were centrifuged at about 2000 rpm for 5 min. The oil was removed and thrown away; the lower portion of the tube was transferred to a cell culture flask containing DMEM with 10% FBS and incubated at 37°C and 5% CO${}_{2}$. At a concentration of 100000 × 8 cells/ml, the medium was collected and kept. In order to prepare the specimen, 1 ml of the sample was cast in a test tube and about 1.5-2 ml of SPAS was slowly added. The tubes were incubated at 37°C for 40 min. Over time, the supernatant was removed gradually and washed with Hams F10 + 2.5% HAS (8).

### Sample preparation by passing through a cumulus column 

The cumulus cells were separated around the oocytes and inserted into the pasteurized pipette; this pipette was then inserted into the suspension sperm prepared by the DGC and swim up method at the end, the sperm passing through the cumulus was collected.

### Ethical considerations 

This experimental study was approved by the Ethics Committee of for Islamic Azad University Science and Research Branch, Tehran, Iran. (Code: IR.IAU.SRB.REC.1397.049). All participants signed informed consent forms.

### Statistical analysis 

Statistical comparisons between groups were performed using ANOVA with Games-Howell and simple independent *t* tests. Also, Pearson's correlation test was applied for comparisons. Data were analyzed using statistical package for social sciences version 15.0, spss lnc, Chicago, Illinois, USA (SPSS) and expressed as mean ± SD. P-values < 0.05 were considered significant.

## 3. Results

In the present study, sperm parameters and the degree of sperm DNA damage in infertile men with asthenoteratozoospermia was investigated in four groups: a control group and groups processed by DGC, SPAS, and cumulus pill method.

### Sperm DNA fragmentation 

**Figure 1:**
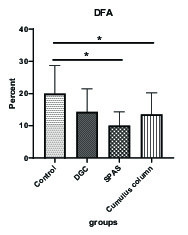
Comparison of the mean of sperm DNA fragmentation in the study groups. Values are expressed as Mean ± SD (ANOVA, Games Howell, and p > 0.05). DGC: Density gradient centrifugation; SPAS: Supernatant products of adipose tissue-derived adult stem cells.

The statistical analysis of the results of the DNA fragmentation test showed a significant reduction in this parameter in the groups processed by the SPAS and the cumulus pill methods compared with the control group (p = 0.021) (Figure 1).

### APAS: Sperm motility

**Figure 2:**
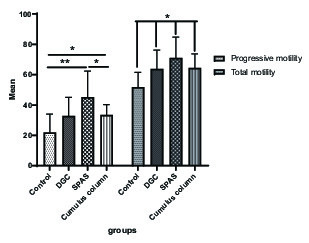
Comparison of the mean total and progressive sperm movement in the study groups (in terms of percentage). Values are expressed as Mean ± SD. Means with the same letter do not differ significantly from each other (ANOVA, Games–Howell test and p > 0.05). DGC: Density gradient centrifugation; SPAS: Supernatant products of adipose tissue-derived adult stem cells.

The sperm motility in all three experimental groups was significantly increased compared to the control group (p = 0.024). The percentage of progressive sperm motility in the SPAS and cumulus pill groups was significantly higher than the control group (p = 0.011), nevertheless, this parameter was not significantly different between the DGC group, the cumulus pill and control groups (p = 0.013). Furthermore, improvement in the progressive sperm motility by SPAS method was statistically more significant than the cumulus pill and the DGC groups (p = 0.033) (Figure 2).

### Sperm morphology and sperm count

**Figure 3:**
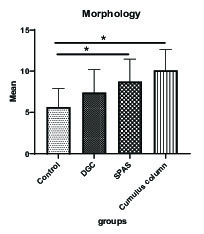
Comparison of the mean normal morphology in study groups (in terms of percentage). Values are expressed as Mean ± SD (ANOVA, Games–Howell, and p > 0.05). DGC: Density gradient centrifugation; SPAS: Supernatant products of adipose tissue-derived adult stem cells.

**Figure 4:**
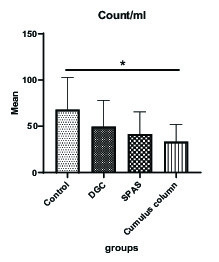
Comparison of the mean sperm count (million/ml) in study groups. Values are expressed as Mean ± SD (ANOVA, Games–Howell, and p > 0.05). DGC: Density gradient centrifugation; SPAS: Supernatant products of adipose tissue-derived adult stem cells.

The mean normal sperm morphology in SPAS and cumulus pill groups was significantly higher than the control group (p = 0.002). In addition, the improvement in the normal morphology by cumulus pill method was statistically more significant than the DGC group (p = 0.01). On the other hand, the cumulus method compared to both the SPAS and DGC methods had a more efficient morphology, although this superiority was statistically significant only in comparison with the DGC method (p = 0.021) (Figure 3). Besides, the statistical analysis of the results in relation to the sperm count indicated a significant decrease in this parameter in the cumulus pill and SPAS groups compared to the control group (p = 0.01) (Figure 4).

## 4. Discussion 

In this study, semen samples of 20 infertile men with asthenoteratozoospermia were compared using three methods: DGC, cumulus pill, and SPAS.

Our results showed that the percentage of total sperm motility was significantly higher in all three groups than the control group (p ≤ 0.05), as expected. Considering the nature of the DGC method, based on the separation of sperm, due to the difference in the density between dead and surviving sperm, the increase in total motility in this group can be justified. These findings are consistent with the findings of Rijsdijk and Franken study in 2007 (9). However, it has been suggested that the mechanical strength of the cumulus matrix may affect the motor's sperm model (10).

According to the results of the present study, although a significant increase in the percentage of sperm motility with SPAS and the cumulus column was shown in comparison with the control group, the comparison of the mean of these two parameters in the SPAS and the cumulus column groups was statistically in favor of the SPAS method (Figure 2). In the SPAS method, it is possible to highlight the contents of the mesenchymal stem cells, including insulin-like growth factor (IGF), hepatocyte growth factor (HGF), and fibroblast growth factor (FGF). Selvaraju and co-workers reported in 2012 that the change in levels of IGF-I in the plasma seminal can affect the development, maturation, and movement of spermatozoa (11). The presence of high levels of HGF in the distal epididymis suggests that this factor induces the increases of sperm motility (12). In addition, it has been shown that FGF receptors 1,2,3,4 are expressed in human testicles and sperm, and their position is limited to the acrosome and flagella membranes. The findings also revealed that several kinases of the FGF/FGFR-signaling pathways (especially ERK and PI3K/Akt) are involved in maintaining movement, capacity-building, acrosome exocytosis, and sperm survival (12, 13).

Our results showed that the SPAS and cumulus column groups were significantly affected by sperm count. Statistical analysis of the results of the sperm morphology in different groups indicated that the SPAS and the cumulus column groups were almost identical in the isolation of normal morphology sperm.

Since one of the defects of chromatin compaction is DNA fragmentation, it can result in incorrect chromatin condensation and cause DNA damage in the reproductive system (7).

In our study, there was a significant decrease in DNA fragmentation in the cumulus column and the SPAS groups compared with the controls. The mechanisms involved in the rearrangement of sperm chromatin are still not fully understood, however, it is assumed that these changes begin at capacity, possibly by reducing the disulfide bridges that reduces chromatin compression and allows re-orientation to histones (10).

A clinical evidence suggests that sperm DNA damage can be considered unfavorable for reproductive results. Investigating the integrity of the sperm DNA can help with the selection of sperm with minimal damage for use in ART.The importance of damage to the DNA of the sperm nucleus on the extent of fertilization is still controversial, but there is some agreement about its negative effects on embryo development and pregnancy rates. In floating wash methods, good sperm are obtained, while DGC is usually used to retrieve the number of moving sperm more than the sample with poor parameters (14).

By using the cumulus column technique, sperm that successfully pass an important physiological stage of the inclination cascade, including capacity and acrosome reactivity, are considered to be without risk factors and are commonly used in the selection of spermatozoa. They are selected on the basis of the appearance parameters (13).

As discussed in a previous study, one of the anomalies that may exist in the sperm, and ultimately lead to irreparable damage to the development of the fetus is the structure of the sperm (14). A new method has recently been introduced, that is, SPAS. It has been shown that the contents of this environment, including growth factors and various cytokines such as interleukin 6, VEGF, Interleukin 8, Granulocyte-colony stimulating factor, Stem cell factor, IGF, HGF, Interleukin 15, Interleukin 10, NGF, PDGF–bb, and BFGF not only improve movement parameters but also surprisingly reduce the amount of DNA fragmentation (12, 8).

## 5. Conclusion 

The comparison of the three methods of sperm preparation showed that the sperm separated by the SPAS method had the lowest DNA fragmentation and the highest sperm motility. However, the number of sperm and the normal morphology of the cumulus column worked better, which is probably due to the nature of this method and the interaction between the sperm and the cumulus cells.

Therefore, we suggest that the method used in the separation and preparation of sperm be used according to the therapeutic purpose.

##  Conflict of Interest

The authors declare that they have no conflict of interest.
